# Metabolic Rewiring of Kynurenine Pathway during Hepatic Ischemia–Reperfusion Injury Exacerbates Liver Damage by Impairing NAD Homeostasis

**DOI:** 10.1002/advs.202204697

**Published:** 2022-10-30

**Authors:** Bowen Xu, Peng Zhang, Xiaolong Tang, Shiguan Wang, Jing Shen, Yuanwen Zheng, Chao Gao, Ping Mi, Cuijuan Zhang, Hui Qu, Shiyang Li, Detian Yuan

**Affiliations:** ^1^ Department of Biochemistry and Molecular Biology School of Basic Medical Sciences Cheeloo College of Medicine Shandong University Jinan Shandong 250012 China; ^2^ Department of General Surgery Qilu Hospital of Shandong University Jinan Shandong 250012 China; ^3^ Advanced Medical Research Institute Shandong University Jinan Shandong 250012 China; ^4^ Department of Hepatobiliary Surgery Shandong Provincial Hospital Affiliated to Shandong First Medical University Jinan Shandong 250117 China; ^5^ Department of Hepatobiliary Surgery General Surgery Qilu Hospital Cheeloo College of Medicine Shandong University Jinan Shandong 250012 China; ^6^ Institute of Pathology and Pathophysiology School of Basic Medical Sciences Cheeloo College of Medicine Shandong University Jinan Shandong 250012 China; ^7^ Department of Gastroenterology Qilu Hospital Cheeloo College of Medicine Shandong University Jinan Shandong 250012 China

**Keywords:** hepatic ischemia–reperfusion, kynurenine pathway, metabolic rewiring, nicotinamide adenine dinucleotide (NAD), redox

## Abstract

Hepatic ischemia–reperfusion (IR) injury remains a common issue lacking effective strategy and validated pharmacological targets. Here, using an unbiased metabolomics screen, this study finds that following murine hepatic IR, liver 3‐hydroxyanthranilic acid (3‐HAA) and quinolinic acid (QA) decline while kynurenine and kynurenic acid (KYNA) increase. Kynurenine aminotransferases 2, functioning at the key branching point of the kynurenine pathway (KP), is markedly upregulated in hepatocytes during ischemia, shifting the kynurenine metabolic route from 3‐HAA and QA to KYNA synthesis. Defects in QA synthesis impair de novo nicotinamide adenine dinucleotide (NAD) biosynthesis, rendering the hepatocytes relying on the salvage pathway for maintenance of NAD and cellular antioxidant defense. Blocking the salvage pathway following IR by the nicotinamide phosphoribosyltransferase inhibitor FK866 exacerbates liver oxidative damage and enhanced IR susceptibility, which can be rescued by the lipid peroxidation inhibitor Liproxstatin‐1. Notably, nicotinamide mononucleotide administration once following IR effectively boosts NAD and attenuated IR‐induced oxidative stress, inflammation, and cell death in the murine model. Collectively, the findings reveal that metabolic rewiring of the KP partitions it away from NAD synthesis in hepatic IR pathophysiology, and provide proof of concept that NAD augmentation is a promising therapeutic measure for IR‐induced liver injury.

## Graphical abstract

Kyat2‐mediated metabolic rewiring of the kynurenine pathway partitions it away from de novo nicotinamide adenine dinucleotide (NAD) biosynthesis in hepatic ischemia–reperfusion injury. Single intraperitoneal dose of nicotinamide mononucleotide post‐reperfusion effectively boosts NAD and reduces liver damage, providing proof of concept that replenishing NAD pools may be a promising and low‐cost strategy against hepatic ischemia–reperfusion injury.

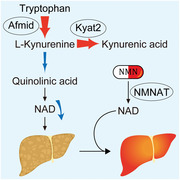

## 1 Introduction

Hepatic ischemia–reperfusion (IR) injury, a serious complication that occurs during liver transplantation, liver resection, and hemorrhagic shock, remains a major challenge adversely affecting patient outcome. Clinical therapies targeting hepatic IR injury mainly rely on antioxidants or anti‐inflammatory drugs. Unfortunately, outcomes are always unsatisfied due to poor pharmacokinetics and serious untoward effects. Ischemic preconditioning seems to be the only promising strategy in clinical practice,^[^ ^1^ ^]^ but this procedure is only applicable to patients with long ischemia time and small hepatectomy volume.^[^ ^2^ ^]^ No validated pharmacological approaches to date have yet been approved for the treatment. Thus, identification of more efficient and clinically compatible strategies against hepatic IR injury is urgently required.

Oxidant stress has long been considered to be attribute to the acute reoxygenationin due to blood reperfusion, while ischemia‐induced acute oxygen blocking, glycogen consumption, and ATP depletion appear to be tolerated by the liver.^[^ ^3^ ^]^ Accordingly, marked metabolic change involves in hepatic reperfusion stage.^[^ ^4^, ^5^ ^]^ However, recent studies have shown that oxidative stress occurs as early as in liver ischemia. A lipidomics analysis report indicates that the arachidonate 12‐lipoxygenase (ALOX12) is markedly elevated in the hepatocytes during ischemia, promoting the accumulation of 12‐hydroxyeicosatetraenoic acid (12‐HETE) and inflammatory that aggravates liver injury.^[^ ^5^ ^]^ So far, the changes in the global metabolite level during hepatic ischemia stage have not been explored.

Nicotinamide adenine dinucleotide (NAD), an essential coenzyme in redox reactions, making it central to energy metabolism.^[^ ^6^ ^]^ NAD also acts as a substrate of nonredox enzymes,^[^ ^7^, ^8^ ^]^ including sirtuin deacetylases (SIRTs), cellular surface cyclic ADP ribose hydrolase CD38, and poly(ADP‐ribose) polymerases (PARP). NAD biosynthesis is governed by at least three pathways,^[^ ^9^ ^]^ namely, de novo synthesis pathway from tryptophan, Preiss–Handler (PH) pathway from nicotinic acid, and salvage pathway from nicotinamide or nicotinamide riboside. In mammalian cells, NAD plays a crucial role in metabolism, DNA repair, epigenetic regulation, senescence, and immunoregulation.^[^ ^10^, ^11^ ^]^ Disruption of cellular NAD homeostasis involves in a lot of diseases, such as aging, obesity, diabetes, alcoholic or nonalcoholic liver disease, and also IR‐related organ injury, such as heart, brain, and kidney.^[^ ^6^, ^7^, ^10^, ^12^, ^13^, ^14^ ^]^ For example, it has been demonstrated that over activation of CD38 leads to NAD depletion in cardiac ischemic injury.^[^ ^15^ ^]^ Genetic or pharmacological blockade of CD38 can notably rescue the outcome. Protein acetylation due to the inhibition of sirtuins, is the reason for cerebral ischemia‐induced injury.^[^ ^16^ ^]^ In acute kidney injury (AKI), de novo NAD biosynthesis and salvage pathways are blocked due to the restricted expression of rate‐limiting enzymes quinolinate phosphoribosyltransferase (QPRT) and NAM phosphoribosyltransferase (NAMPT).^[^ ^12^ ^]^ Similar to kidney but unlike the other organs, livers actively make NAD via de novo synthesis and release NAM into the blood, supporting the rest organs for NAD biosynthesis.^[^ ^17^ ^]^ However, whether and how the NAD biosynthesis is changed in the pathophysiology of hepatic IR injury remain not fully investigated.

Here, we report that kynurenine pathway (KP) is reprogrammed during liver ischemia in the murine hepatic IR model, skewing kynurenine metabolism toward kynurenic acid (KYNA) synthesis. KP reshuffling compromised‐de novo NAD synthesis is partially responsible for IR‐induced liver damage. Strikingly, NAD boosting by nicotinamide mononucleotide (NMN) replenishment could protect against liver damage by alleviating oxidative stress, inflammation, and cell death. Collectively, we identify an important role of NAD in promoting resistance against hepatic IR damage, and provide proof of concept that NAD augmentation might be a therapeutic option for liver IR treatment.

## 2 Results

### 2.1 Metabolic Rewiring of the Kynurenine Pathway in Liver Ischemia

**Figure 1:**
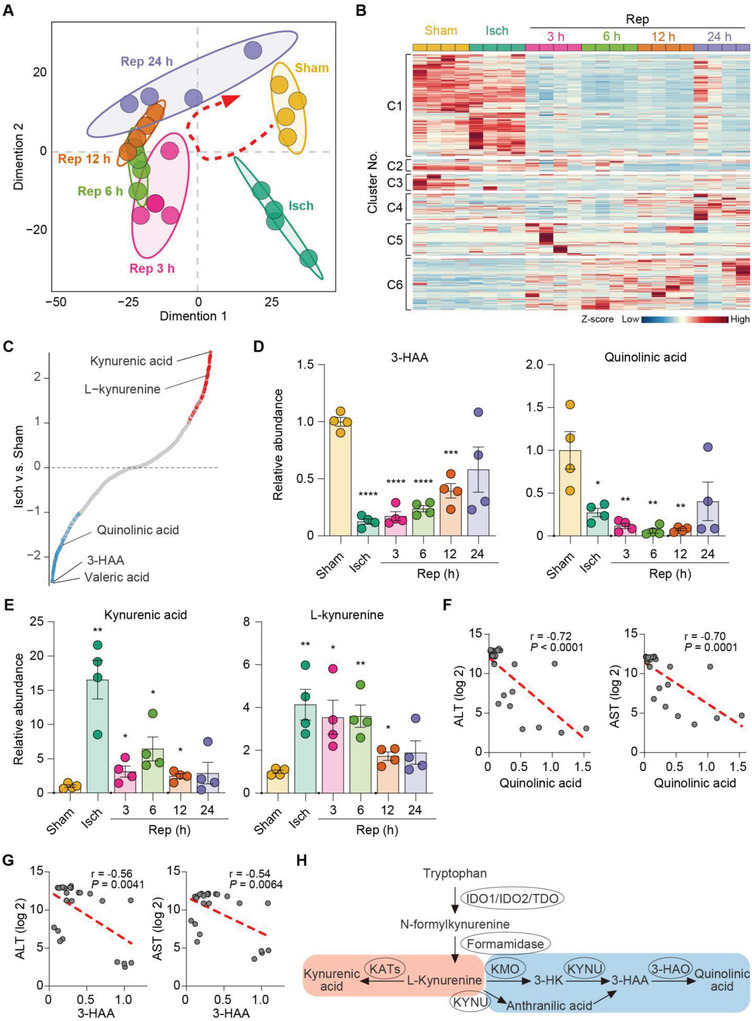
Metabolic rewiring of the KP during hepatic IR injury. A) Global sample distribution profiles and relationships analyzed by the principal component analysis. *n* = 4 mice for each group. B) Six distinct clusters and the expression trends for each period of hepatic IR determined by *k*‐means clustering analyses with the normalized metabolite levels of biological replicates using the *Z*‐score transformation method. C) Relative abundance of individual metabolite between ischemia and sham groups. l‐kynurenine, kynurenic acid, 3‐HAA, and QA were highlighted. D) Relative abundance of 3‐HAA and quinolinic acid in the livers of mice subjected to 90‐min ischemia followed by reperfusion for the indicated periods. *n* = 4 mice for each group. E) Relative abundance of kynurenic acid and l‐kynurenine in the livers of mice subjected to 90‐min ischemia followed by reperfusion for the indicated periods. *n* = 4 mice for each group. F) Scatter plot depicting the correlation between the levels of quinolinic acid with ALT and AST levels in the livers subject to IR. G) Scatter plot depicting the correlation between the levels of 3‐HAA with ALT and AST levels in the livers subject to IR. H) Diagram of the kynurenine pathway showing reduced 3‐hydroxyanthranilic acid and quinolinic acid synthesis and concurrent enhanced kynukenic acid synthesis during hepatic IR injury. For statistical analysis in (D) and (E), one‐way ANOVA with Tukey's post hoc test was used. The correlations were evaluated with Spearman's test in (F) and (G). *P < 0.05, **P < 0.01, ***P < 0.001, ****P < 0.0001. Data are presented as the mean ± SEM.

To investigate the alteration of metabolic profiles during hepatic IR injury, we performed an unbiased metabonomics analysis using liver tissue samples from mice without vascular occlusion and mice after 90 min ischemia followed by reperfusion for 0, 3, 6, 12, and 24 h (Figure S1A, Supporting Information). Based on principal component analysis and a recursive hierarchical clustering scheme analyses, we observed that the IR‐related subgroups displayed a distinct classification compared to the sham group, which were mutually separated in a time‐dependent manner (Figure 1A; Figure S1B, Supporting Information). Under the k‐means clustering analyses with the normalized metabolite levels of biological replicates, the metabolome reprogramming during IR were well visualized into six distinct clusters associated to their close expression trends (Figure 1B), reflecting an unique metabolic alteration in the hepatic IR groups when compared with the sham group. Valeric acid, a bacterially produced metabolite, was the most downregulated metabolite in the ischemic stage, reflecting the direct consequence of portal venous occlusion. We noticed that 3‐hydroxyanthranilic acid (3‐HAA) produced by catalytic hydrolysis of kynurenine through kynureninase and kynurenine 3‐monooxygenase, was the second most downregulated hit during ischemia, while KYNA was the most upregulated metabolite (Figure 1C; Figure S1C, Supporting Information). Consistently, QA, downstream of 3‐HAA, was also strongly declined (Figure 1C; Figure S1C, Supporting Information). 3‐HAA and QA are intermediate metabolites of the KP.^[^ ^18^ ^]^ Decline of 3‐HAA and QA and increased levels of kynurenine and KYNA indicated a rewiring of the KP during ischemia (Figure 1C,E and Figure S1C, Supporting Information). Moreover, this metabolic rewiring persisted throughout the IR process, as reflected by the constant reduced 3‐HAA and QA levels and elevated kynurenine and KYNA levels during the reperfusion stage (Figure 1D,E). Both 3‐HAA and QA levels displayed a strong correlation with serum aminotransferase levels in the ischemic liver (Figure 1F,G), indicative of a remarkable relationship between metabolic rewiring of the KP and IR‐induced hepatocytes damage. Overall, these data indicated that hepatic ischemia induces a significant metabolic skewing on KP from 3‐HAA and QA toward KYNA synthesis (Figure 1H).

### 2.2 Upregulation of Afmid and Kyat2 Was Temporally in Sync with the Metabolic Skewing of Kynurenine Pathway

**Figure 2:**
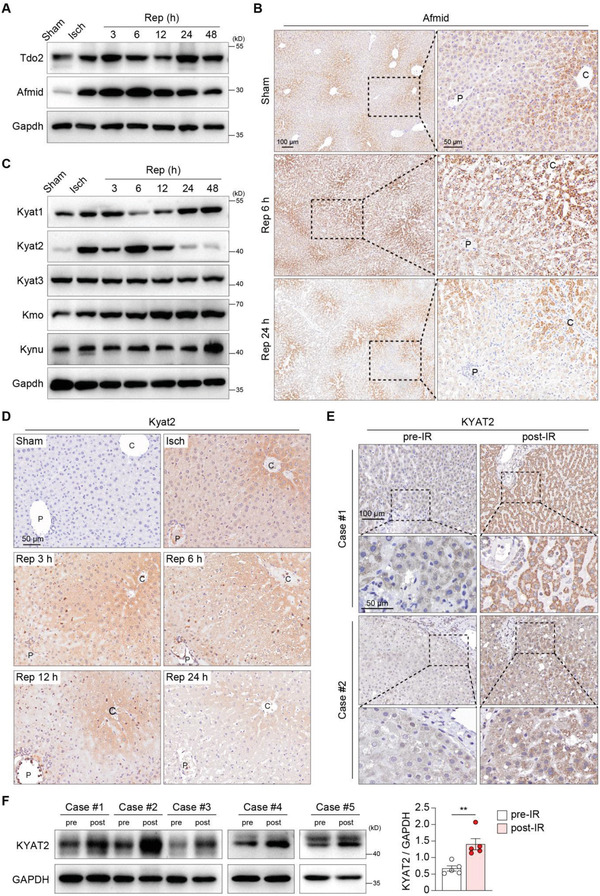
Afmid and Kyat2 are dramaticlly upregulated in the post‐ischemic liver. A) Immunoblots of the indicated proteins in livers of mice subjected to 90 min of ischemia and subsequent reperfusion for the indicated durations. Gapdh served as a loading control. B) Representative IHC staining of Afmid in liver sections from mice in the sham group (at baseline) or at 6 and 24 h after reperfusion. C) Immunoblots of the indicated proteins in livers of mice subjected to 90 min of ischemia and subsequent reperfusion for the indicated durations. Gapdh served as a loading control. D) Representative IHC staining of Kyat2 in livers of mice subjected to 90 min of ischemia and subsequent reperfusion for the indicated durations. E) Representative IHC of KYAT2 in the liver sections of individuals subjected to hepatic IR surgery. F) KYAT2 protein levels determined by western blot in the livers of individuals subjected to hepatic IR surgery. Pre, pre‐IR; post, post‐IR. GAPDH served as a loading control. Bar graph represents mean ± SEM. Paired Student's *t*‐test was used. **P < 0.01.

To study the regulatory mechanism on the metabolic reprogramming of KP during hepatic IR, we measured the expression of key enzymes implicated in KP throughout liver IR process. KP is initiated by either intrahepatic tryptophan 2,3‐dioxygenase (Tdo2), or extrahepatic indoleamine 2,3‐dioxygenase (Ido1 and Ido2), followed by arylformamidase (Afmid) catalyzing N‐formylkynurenine to l‐kynurenine (Figure 1H). Although we did not observe consistent changes in the protein levels of Tdo2 throughout hepatic IR process, dramatic upregulation of Afmid at both mRNA and protein levels was evident from the ischemia stage (Figure 2A; Figure S3A, Supporting Information). Immunohistochemistry (IHC) staining of Afmid further revealed that expression of Afmid was largely restricted in the peri‐central hepatocytes in livers from the sham‐operated group (Figure 2B). Zonal expression of Afmid expanded to the mid‐lobular and peri‐portal regions after hepatic IR, with Afmid expression peaking at 6 h post‐reperfusion (Figure 2B). After 24 h post‐reperfusion, the regional expression of Afmid was restored (Figure 2B). Clearly, this robust upregulation of Afmid during IR process may account for the robust elevation of kynurenine as shown above (Figure 1C,E).

Next, we examined the expression of enzymes downstream of kynurenine, including Kmo and kynurenine amino transferases (Kats, including Kyat1, Kyat2, and Kyat3), which, respectively, contributed to kynukenic acid and 3‐hydroxykynurenine (3‐HK) anabolism.^[^ ^18^ ^]^ Interestingly, we discovered that only Kyat2 expression was strongly increased in ischemic liver, with low or negligible expression in control liver, as assayed by immunoblotting (Figure 2C). By contrast, slight upregulation of Kmo and negligible changes of Kyat1 and Kyat3 were observed during IR compared to sham treatment (Figure 2C; Figure S2A,B, Supporting Information). IHC further confirmed that the dynamic change of Kyat2 expression throughout the reperfusion stage (Figure 2D). Elevated expression of Kyat2 returned to normal level after 24 h post‐reperfusion (Figure 2C,D). We also examined expression levels of KYAT2 in liver tissue samples from patients who underwent liver resection surgery owing to hepatocellular carcinoma or hepatic cysts, and observed significantly increased protein levels of KYAT2 compared with those in pretreatment groups (Figure 2E,F).

Unlike *Afmid* that was upregulated at transcriptional level (Figure S3A, Supporting Information), mRNA levels of *Kyat2* were largely unchanged in ischemic liver (Figure S3A, Supporting Information). Translational control of protein synthesis in response to environmental stress involves phosphorylation of the *α* subunit of eukaryotic initiation factor 2 (eIF2*α*), which inhibits global protein synthesis while promoting translation of genes involved in the stress response.^[^ ^19^ ^]^ Given that eIF2*α* phosphorylation is implicated in IR injury in several organs including the liver,^[^ ^20^ ^]^ we tested whether upregulation of Kyat2 was related to eIF2*α* phosphorylation. Indeed, p‐eIF2*α* was elevated in ischemic liver, in parallel with the upregulation of Kyat2 (Figure S3B–E, Supporting Information). Moreover, inhibition of eIF2*α* phosphorylation by ISRIB attenuated Kyat2 upregulation in ischemic liver, implying that p‐eIF2*α*‐mediated translational control is, at least in part, responsible for Kyat2 expression induced by hepatic IR (Figure S3B–E, Supporting Information).

Taken together, these data suggest that Kyat2 may play a leading part in kynukenic acid synthesis during IR process, resulting to an abundant consumption of kynurenine and relative deprivation of the availability of kynurenine for QA anabolism branch.

### 2.3 NAD Deprivation Paralleled with Lipid Peroxidation in the Post‐Ischemic Liver

**Figure 3:**
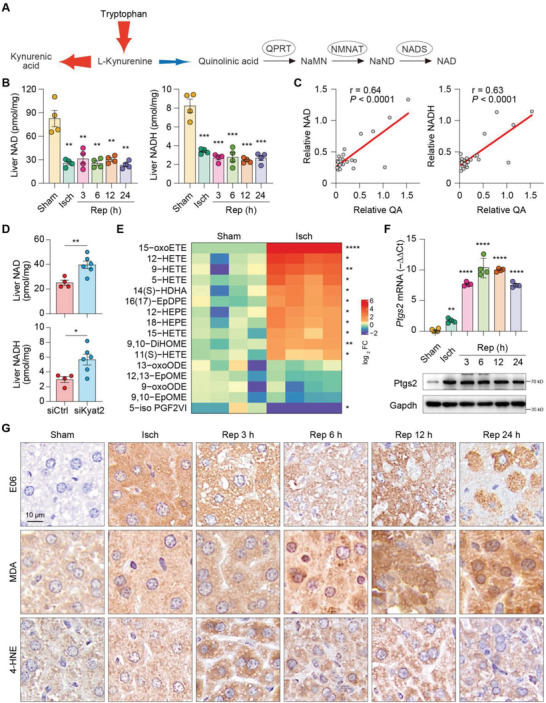
NAD deprivation paralleled with lipid peroxidation in post‐ischemic liver. A) Diagram of the de novo NAD biosynthesis pathway from tryptophan. B) Liver NAD and NADH content in mice subjected to 90 min of ischemia and subsequent reperfusion for the indicated durations. *n* = 4 mice per group. C) Scatter plot depicting the relevance of liver NAD and NADH content and quinolinic acid content in livers subjected to hepatic IR surgery. D) Liver NAD and NADH contents from mice with 90‐min ischemia after siKyat2 or siCtrl treatment. *n* = 4–6 mice per group. E) Heatmap of UPLC‐MS/MS detection of a penal of oxidized fatty acids in ischemic livers and sham‐operated controls. *n* = 4 mice per group. F) mRNA and protein levels of Ptgs2 in livers from mice subjected to hepatic IR surgery. *n* = 4 mice per group. Gapdh served as a loading control. G) Representative IHC staining of E06, malondialdehyde (MDA), and 4‐HNE in the liver sections of mice subjected to hepatic IR surgery. Scale bar, 10 µm. *n* = 4 mice per group. Unpaired Student's *t*‐test was used. **P < 0.01, ***P < 0.001, ****P < 0.0001. Data are presented as the mean ± SEM.

As QA is the fundamental intermediate metabolite for NAD de novo synthesis from tryptophan in liver,^[^ ^18^ ^]^ the direct consequence of inadequate QA synthesis would be the breakdown of de novo NAD biosynthesis (**Figure** **3**A). Indeed, as compared with sham groups, significantly reduced intrahepatic NAD levels were detected in the IR groups, and this downregulation started from the ischemia stage and lasted over the entire reperfusion periods (Figure 3B). In addition, liver NAD levels exhibited strong correlation with the levels of QA, suggesting that the decline of intrahepatic NAD after IR was related to the impaired QA synthesis (Figure 3C).

NAD is an important redox carrier and it serves as a substrate for NAD‐dependent enzymes.^[^ ^7^, ^8^, ^10^ ^]^ Most recently, NAD has been shown to neutralize lipid peroxidation and protect against ferroptosis through FSP1/ubiquinone axis.^[^ ^21^ ^]^ Since ferroptosis emerges to be the main cause of IR‐induced tissue damage in various organs, we asked whether the rewired NAD biosynthesis starting during hepatic ischemia was linked to lipid peroxidation and ferroptosis. Indeed, ultra‐performance liquid chromatography tandem mass spectrometry (UPLC‐MS/MS) detection of a penal of oxidized fatty acids revealed that 11 out of 16 oxidized lipid metabolites were significantly elevated in ischemic livers compared to sham‐operated controls (Figure 3E; Figure S4A, Supporting Information). Consistently, upregulation of Ptgs2, a pharmacodynamic marker of ferroptotic tissues,^[^ ^22^, ^23^ ^]^ was confirmed at both mRNA and protein levels (Figure 3F). Importantly, this upregulation initiated from the ischemia stage, in parallel with the blockade of NAD de novo synthesis (Figure 3B). We next assessed the liver peroxidation level using multiple biomarkers indicating oxidative stress in the liver tissues from mice after hepatic IR insult. IHC staining confirmed phospholipid peroxidation of parenchymal cells in ischemic livers compared with sham group, as determined by E06 that recognized oxidized phospholipids. This trend continued following reperfusion duration, as characterized by diffusing positive staining in the cytoplasm and membrane of hepatocytes (Figure 3G; Figure S4B, Supporting Information). Consistently, malondialdehyde (MDA) and 4‐hydroxynonenal (4‐HNE), two major reactive breakdown products of the lipid peroxides that execute ferroptosis, also upregulated from the ischemia stage and lasted over the reperfusion period (Figure 3G; Figure S4C,D, Supporting Information).

To address whether kyat2 upregulation was causally linked to liver NAD decline and lipid peroxidation, cationic liposome‐encapsulated short interfering RNAs (siRNAs) targeting mouse Kyat2 (siKyat2) were injected intravenously into the mice and hepatic IR was performed 48 h later (Figure S5A, Supporting Information). Kyat2 knockdown in the ischemic livers was confirmed by immunoblotting, with >50% decrease in protein expression compared to ctrl siRNA (siCtrl) (Figure S5B, Supporting Information). Notably, Kyat2 knockdown restored the NAD and NADH levels in the ischemic livers, concomitant with reduced levels of oxidative stress as determined by IHC staining of E06, MDA, and 4‐HNE (Figure 3D; Figure S5C, Supporting Information). Macroscopic examination of the livers 24 h post‐reperfusion revealed less lesions in siKyat2‐treated mice compared to control animals (Figure S5D, Supporting Information). Serum ALT and AST in siKyat2‐treated mice were also reduced than those in the siCtrl group at 24 h post‐IR, indicative of attenuated liver injury (Figure S5E, Supporting Information). Furthermore, we observed reduced immune infiltration as determined by IHC staining of CD45 and Ly6B in siKyat2‐treated livers compared to siCtrl‐treated controls (Figure S5F, Supporting Information). The mRNA levels of proinflammatory factors including *Il1a*, *Il1b*, *Ccl3*, *Ccl2*, *Ccr1*, and *Tgfb* were significantly reduced in the siKyat2‐treated livers compared with those in the siCtrl‐treated livers (Figure S5G, Supporting Information). Taken together, these data indicate that hepatic ischemia‐induced Kyat2 upregulation causes NAD deprivation and lipid peroxidation, which initiates before the abrupt availability of oxygen during reperfusion.

### 2.4 Nampt Inhibition by FK866 Aggravates IR‐Induced Liver Damage

**Figure 4:**
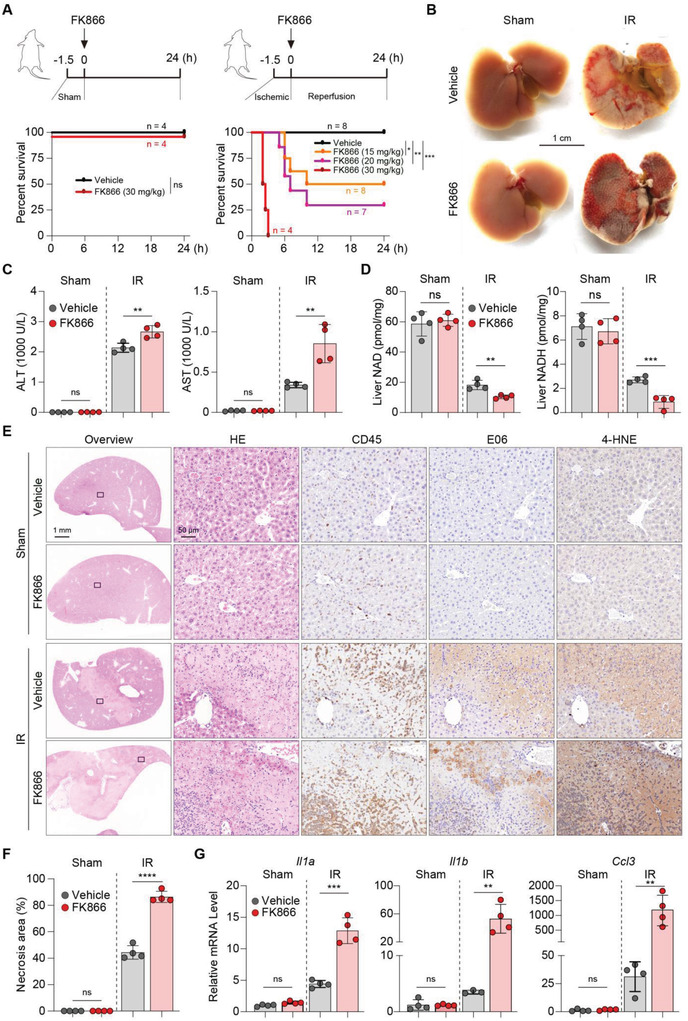
Nampt inhibition by FK866 aggravates IR‐induced liver damage. A) Upper schemes illustrate the protocols of FK866 treatment in the sham‐treated (left) and IR‐treated (right) mice; bottom left, Kaplan–Meier survival curve of mice treated with 30 mg kg^−1^ FK866 or vehicle in the sham group; bottom right, Kaplan–Meier survival curve of mice treated with 15, 20, and 30 mg kg^−1^ FK866 or vehicle in the IR group. B) Representative images of gross morphology of livers from 24 h post‐reperfusion or sham mice treated with 15 mg kg^−1^ FK866 or vehicle. C) Serum ALT and AST levels in livers from 24 h post‐reperfusion or sham mice treated with 15 mg kg^−1^ FK866 or vehicle. D) Liver NAD and NADH contents in sham controls or mice 24 h after hepatic IR. E) HE and IHC staining of CD45, E06, and 4‐HNE in sham and IR‐injured livers treated with FK866 or vehicle. F) Quantification of necrosis area in (E). G) Relative mRNA levels of *Il1a*, *Il1b*, and *Ccl3* in sham and IR‐injured livers treated with FK866 or vehicle. Unpaired Student's *t*‐test was used. *P < 0.05, **P < 0.01, ***P < 0.001, ****P < 0.0001. B–G) *n* = 4 mice per group. Data are presented as the mean ± SEM.

We reasoned that NAD loss may have influenced cell redox state and oxidative‐stressed damage in IR‐stressed livers as NAD plays important roles in the maintenance of cellular redox homeostasis. To that end, we blocked salvage pathway of NAD biosynthesis by intraperitoneal (i.p.) injection of FK866, an inhibitor of Nampt, in the mice after 90 min ischemia or sham treatment. FK866 treatment even at a high dose (30 mg kg^−1^) did not cause mortality in mice without hepatic IR (**Figure** **4**A). We did not observe any macroscopic abnormality in livers from FK866‐treated animals either (Figure 4B). In addition, liver NAD levels were largely unaffected, suggesting that NAD consumption of physiological livers mainly rely on de novo NAD synthesis pathway but not Nampt‐mediated salvage pathway (Figure 4D). We did not find any signs of liver injury, i.e., aggravated liver injury, necrotic morphology, production of pro‐inflammatory cytokine and chemokine, and infiltration of inflammatory cells (Figure 4C–F). These findings are in line with the report that FK866 treatment did not display noticeable effect on NAD content.^[^ ^14^ ^]^

We next tested the effects of FK866 treatment in mice with hepatic IR surgery (Figure 4A). Notably, the FK866‐treated mice exhibited a marked dose‐dependent decrease in mortality, with the 15, 20, and 30 mg kg^−1^ groups demonstrating 50%, 25%, and 0% survival, respectively (Figure 4A). The gross appearance of the liver revealed more severe lesions, characterized by dark complexion and uneven surface (Figure 4B). Contrary to the unaffected levels of NAD in livers without hepatic IR surgery, liver NAD levels after hepatic IR surgery were further decreased upon FK866 treatment (Figure 4D). Serum ALT and AST in FK866‐treated mice were much higher than those in the control group at 24 h post‐IR (Figure 4C). Furthermore, the histological examination demonstrated significantly increased necrotic areas and infiltration of Ly6B‐positive inflammatory cells in FK866‐treated livers compared to vehicle‐treated controls (Figure 4E,F; Figure S7A,B, Supporting Information). mRNA levels of proinflammatory cytokines and chemokines, for example, *Il1a*, *Il1b*, *Tnf*, *Ccl2*, *Ccl3*, and *Ccl4* were significantly increased in the FK866‐treated livers compared with those in the vehicle‐treated livers (Figure 4G; Figure S7C, Supporting Information). By contrast, blocking the Preiss‐Handler pathway by nicotinate phosphoribosyltransferase (Naprt) inhibitor 2‐hydroxynicotinic acid (2‐HNA) had little effect on hepatic IR‐induced liver injury, as evidenced by comparable mortality after surgery, gross liver appearance as well as serum ALT and AST levels between 2‐HNA‐treated and the vehicle‐treated animals (Figure S6A–C, Supporting Information). These results demonstrate that Nampt inhibition by FK866 blocks NAD production and aggravates liver damage under the condition of hepatic IR, but not in the steady‐state condition.

### 2.5 Blocking Lipid Peroxidation Reverses the Lethal Effect of FK866 on Hepatic IR Injury

**Figure 5:**
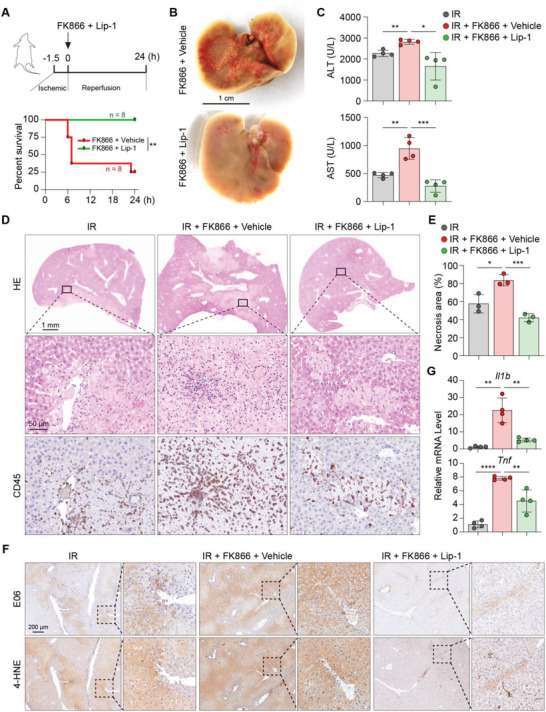
Blocking lipid peroxidation reverses the lethal effect of FK866 on hepatic IR‐injured mice. A) Upper scheme illustrates the experimental procedure; Bottom, Kaplan–Meier survival curve of IR‐injured mice treated as indicated. *n* = 8 mice per group. B) Gross morphology of livers from IR‐injured mice treated as indicated. C) Serum ALT and AST levels in the serum of IR‐injured mice treated as indicated. *n* = 4 mice per group. D,E) HE and IHC staining of CD45 in liver sections from IR‐injured mice treated as indicated (D), and the corresponding quantification of necrosis areas was shown in (E). *n* = 3 mice per group. F) IHC staining of E06 and 4‐HNE in liver sections from IR‐injured mice treated as indicated. G) Relative mRNA levels of *Il1b* and *Tnf* in IR‐injured livers treated as indicated. *n* = 4 mice per group. Unpaired Student's *t*‐test was used. *P < 0.05, **P < 0.01, ***P < 0.001, ****P < 0.0001. Data are presented as the mean ± SEM.

Considering the critical effect of NAD on redox biology, particularly the lipid peroxidation revealed most recently, we examined whether blocking lipid peroxidation using Lip‐1 could suppress the aggravated liver injury by FK866 in the murine hepatic IR model. Lip‐1 was administered together with FK866 immediately after the hepatic IR surgery (**Figure** **5**A). The lethality of FK866 on mice with hepatic IR surgery was fully rescued by Lip‐1 treatment (Figure 5A). Twenty‐four hours post IR surgery, mice co‐treated with FK866 and Lip‐1 displayed bright and smooth gross appearance, decreased liver transaminase and less parenchymal necrosis (Figure 5B–E). Expression of pro‐inflammatory cytokines and hepatic immune cell recruitment were also substantially reduced (Figure 5D,G; Figure S8A,B, Supporting Information). This protective effect of Lip‐1 was associated with diminished cellular oxidative products related to lipid peroxidation, as detected by IHC of E06 and 4‐HNE (Figure 5F). These results suggest that lipid peroxidation due to NAD decline caused by Nampt inhibition, at least partially, responsible for the augmented liver damage in the mouse model of hepatic IR.

### 2.6 Boosting NAD Production Effectively Ameliorates Hepatic IR Injury in Mice

**Figure 6:**
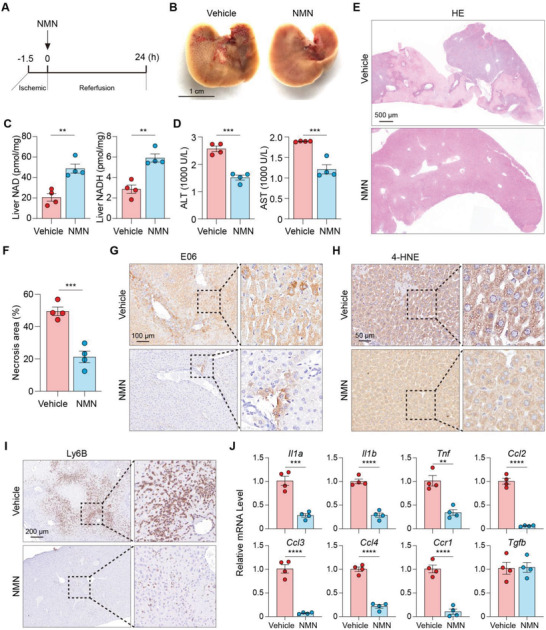
NMN administration ameliorates hepatic IR injury in mice. A) Schemes illustrate the experimental procedure. B–D) Representative images of gross morphology of livers after 90‐min ischemia and 24 h reperfusion with NMN (500 mg kg^−1^) or vehicle, the corresponding serum ALT and AST levels, and liver NAD and NADH contents, respectively. E) H&E staining showing NMN ameliorates IR‐induced liver injury. F) Quantification of necrosis areas. G–I) IHC staining of E06, 4‐HNE, and Ly6B, respectively, in liver sections from IR‐injured mice treated with NMN or vehicle. J) Relative mRNA expression levels for the indicated genes in IR‐injured livers treated with NMN or vehicle. Unpaired Student's *t*‐test was used. **P < 0.01, ***P < 0.001, ****P < 0.0001. B–J) *n* = 4 mice per group. Data are presented as the mean ± SEM.

NMN administration was highly effective in provoking liver NAD production after hepatic IR compared to vehicle‐treated controls (**Figure** **6**A,B,D). Consistent with the restoration of liver NAD levels, NMN supplementation significantly decreased serum transaminase levels and cell death (Figure 6C–F), suggesting that IR‐induced liver dysfunction was attenuated. This effect was accompanied by lower levels of oxidative stress, as evident by the remarkable reduction of both E06 and 4‐HNE staining in NMN‐treated livers at 24 h after reperfusion (Figure 6G,H). Consistently, hepatic IR‐induced inflammatory responses, including infiltration of inflammatory cells, production of cytokine and chemokine, were all significantly attenuated by NMN administration (Figure 6I,J; Figure S9A,B, Supporting Information). These results suggest that boosting NAD production is effective in protecting livers against IR‐induced tissue damage.

## 3 Discussion

Here, we uncover that metabolite constituents and contents have changed markedly during liver ischemia using an unbiased metabolomics screen analysis. We demonstrate that KP is reprogrammed in mice livers with hepatic IR surgery, shifting KYN metabolism toward KYNA generation, which is due to the upregulation of the metabolic enzymes Afmid and Kyat2 in sync temporally. Our data demonstrate mice livers under IR injury mainly rely on Nampt‐mediated salvage pathway for NAD supplement, as NAD produced de novo from tryptophan is limited. Furthermore, NAD deprivation is partially responsible for lipid peroxidation, inflammatory response, and cell death of IR insulted livers, and this damage could be rescued via using NMN, suggesting that NMN for NAD boosting might serve as an attractive therapeutic candidate for hepatic IR treatment.

The KP was identified from non‐biased metabolomic screening in our study. Together with the findings that metabolic reprogramming of the KP regulates renal IR injury^[^ ^24^ ^]^ by Torosyan et al., and cardiac IR injury^[^ ^25^ ^]^ by Olenchock et al., these independent studies suggest that the KP plays a central and perhaps unifying role in IR‐induced tissue injury. Kynurenine can be converted to either KYNA by KATs or GOT2, or to anthranilic acid by KYNU, or to QA for NAD biogenesis. KYNA serves as an antagonist for the *α*7 nicotinic acetylcholine (*α*7nACh) and N‐methyl‐d‐aspartate (NMDA) receptors and it is an agonist for aryl hydrocarbon receptor (AhR).^[^ ^25^ ^]^ Studies have previously shown that KYNA was tissue protective in models of myocardial infarction, cerebral and renal ischemia by antagonizing the glycine binding site of NMDA receptors or activating AhR, which could cause immunosuppression after binding with dioxin.^[^ ^25^, ^26^ ^]^ A previous study showed that KYNA increased energy utilization by activating G protein‐coupled receptor 35 (GPR35), which stimulated lipid metabolism, thermogenic, and inflammatory inhibition in adipose tissue.^[^ ^27^ ^]^ Interestingly, GPR35 is a hypoxia inducible factor (HIF) target that it could be induced during cardiac remodeling.^[^ ^28^ ^]^

To date, which targets or pathways can be used to effectively and safely restore NAD homeostasis are still under investigation. Our study highlights the importance of studying the precise mechanisms underlying the decline in NAD levels in specific diseases, which determine the translatability of NAD to promote treatment in humans. NAD homeostasis imbalance involves divergent mechanisms. For example, de novo NAD synthesis pathway plays a critical role in AKI,^[^ ^12^ ^]^ in which the activity of rate‐limiting enzyme QPRT is particularly crucial for AKI resistance. It has been documented that the blockade of axonal transport of nicotinamide mononucleotide adenylyltransferase 2 (NMNAT2), an enzyme of Preiss–Handler pathway, leads NAD depletion, which might be the reason for neuronal disorders.^[^ ^29^ ^]^ Another example is that the *α*‐amino‐*β*‐carboxymuconate‐*ε*‐semialdehyde decarboxylase (ACMSD), mainly detected in liver and kidney in humans, determines the proportion of ACMS able to undergo cyclization and form the NAD precursor QA.^[^ ^30^ ^]^ Beneficial effects of selective ACMSD inhibitors have been demonstrated in animal models of non‐alcoholic fatty liver disease (NAFLD) and AKI.^[^ ^31^ ^]^ Here we found that de novo NAD synthesis pathway was blocked due to QA decline during hepatic IR, and salvage pathway mainly provided NAD and thus resistance against liver IR damage. However, physiological livers mainly depend on de novo synthesis pathway for NAD requirement, which has been confirmed by our FK866 experiments on mice livers with sham treatment. Therefore, our data and previous studies not only point to the potent plasticity of NAD metabolic pathway under physiological conditions, but also suggest that monitoring the enzymatic activities of the key players in the KP may provide insights into the pathological mechanisms for various diseases characterized by dysregulation of NAD metabolism.

Depletion of NAD pool is closely associated with degenerative diseases and aging,^[^ ^6^ ^]^ in which ferroptosis is heavily involved. Most recently, NAD(P)H has been uncovered to be engaged in the regeneration of CoQ_10_ that traps lipid peroxyl radicals, placing CoQ_10_–NAD(P)H pathway in a stand‐alone system in parallel with GPX4 to inhibit phospholipid peroxidation and ferroptosis.^[^ ^32^ ^]^ Our study demonstrates that the early changes in metabolism during ischemia involve rewiring of KP, which acts as a key initiator of phospholipid peroxidation and liver damage during subsequent reperfusion. In addition, it is worth mentioning that although NMN replenishment and Lip‐1 treatment are comparable in mitigating liver redox damage, NMN is superior to Lip‐1 in depressing inflammatory levels confirmed by our qPCR data of chemokines, which is in line with previous reports that NAD not only participates in cellular redox state regulation, but also functions on regulation of immune cells.^[^ ^11^ ^]^ Besides acting as a coenzyme for redox reactions, NAD is also an essential cofactor for hundreds of nonredox NAD‐dependent enzymes,^[^ ^8^ ^]^ such as sirtuins, PARPs, CD38, and cADPRSs. Future research warrants detailed investigations into the redox‐independent mechanisms of NAD in liver ischemic injury.

In conclusion, our study delineates an early induction of Kyat2 during the ischemic stage as a potent metabolic driver for hepatic IR injury. Kyat2‐mediated metabolic reprogramming of the KP involves KYNA accumulation and decline of 3‐HAA and QA synthesis in the ischemic liver, which acts as a key initiator of the subsequent NAD deprivation, lipid peroxidation, inflammation and liver injury during reperfusion. As there is currently no effective therapy for preventing hepatic IR injury, the beneficial effects of single intraperitoneal dose of NMN post‐reperfusion indicate that replenishing NAD pools may be a promising and low‐cost strategy to improve clinical outcomes in patients with IR‐induced liver damage.

## 4 Experimental Section

### Human Samples

All of the experiments performed including human samples were approved by the Ethics Committee of Shandong University (Document No. ECSBMSSDU2021‐1‐19). Liver tissue donors were patients who underwent liver resection surgery owing to hepatocellular carcinoma or hepatic cysts. These samples were collected from tumor‐ and cyst‐free liver tissue from the individuals at two time points: before ischemia (pre‐IR group) and after reperfusion (post‐IR group). Informed consent forms were signed by all donors or their families. All samples were used only to achieve the experimental objective.

### Animals

All animal procedures and experiments were performed in accordance with the policies of the Institutional Animal Care and Use Committee of the Shandong University (Document No. ECSBMSSDU2021‐2‐79). In consideration of female mice would be sensitive to metabolic environment and stress reaction, the male mice as test subjects were used in the present study. Male wild‐type mice (6–8 weeks of age, 20 ± 2 g) on the C57BL/6 background were purchased from Charles River Laboratories (Beijing, China). All mice were housed in well‐ventilated cages under a constant temperature (23  ±  2 °C) and humidity (50%–60%), with a 12 h–12 h light–dark cycle in a pathogen‐free controlled environment. Food and water were unrestricted access.

### Mouse Liver IR Injury Model

An established partial (70%) liver warm ischemia model^[^ ^33^ ^]^ was used in this study. In brief, mice were first anesthetized with pentobarbital sodium at 50 mg kg^−1^ and then a midline laparotomy was performed. Then, noninvasive microvascular forceps were used to block the blood supply to the left lobe and middle lobe. A successful ischemia treat was indicated by bleaching of the ischemic liver lobes. After ischemia for 90 min and reperfusion for 3, 6, 12, and 24 h, the mice were sacrificed, and liver samples and serum were collected for follow‐up examination. The mice in the sham group underwent the same procedure, but did not have their blood vessels operated on. The heating pads were used to maintain the body temperature throughout the IR procedure.

### Reagents

NMN was purchased from Topscience (T4721) and administered as 500 mg kg^−1^ by intraperitoneal injections for mice with IR protocol. FK866 (T2644, Topscience) was administered as 15, 20, or 30 mg kg^−1^ by intraperitoneal injection for the mice according to the experiment designs. Mice were injected intraperitoneally with 10 mg kg^−1^ Lip‐1 (S7699, Selleck) or vehicle control in our experiments.

### In Vivo siRNA Delivery

The in vivo‐jetPEI (Polyplus Inc.) was used for siRNA delivery according to the manufacturer's instructions. siRNA sequences used in this study are summarized in Key Resources Table.

### Untargeted Metabolomics Analysis

Metabolite extraction was primarily performed according to previously reported methods.^[^ ^34^ ^]^ In brief, 25 mg tissues were used for extraction by directly adding 800 µL of precooled extraction reagent (methanol:acetonitrile:water (2:2:1, v/v/v)), internal standards mix was added for quality control of sample preparation. After homogenizing for 5 min, samples were sonicated for 10 min and incubated at −20 °C for 1 h, and then centrifuged for 15 min at 25 000 rpm at 4 °C. The supernatant was transferred for vacuum freeze drying. The metabolites were resuspended in 600 µL of 70% acetonitrile and sonicated for 10 min at 4 °C. After centrifuging for 15 min at 25 000 rcf, the supernatants were transferred to autosampler vials for LCMS analysis. A quality control (QC) sample was prepared by pooling the same volume of each sample to evaluate the reproducibility of the whole LC‐MS analysis. This experiment used a Waters 2D UPLC (waters, USA) tandem Q Exactive high‐resolution mass spectrometer (Thermo Fisher Scientific, USA) for separation and detection of metabolites.

### UPLC–MS/MS Analysis

Liver contents of oxidized fatty acids were examined by UPLC‐MS/MS analysis. In brief, 20 mg tissues were homogenized using a ball mill (30 HZ) for 20 s and centrifuged at 3000 rpm for 30 s at 4 °C. 1 mL of lipid extraction solution containing internal standard (methyl *tert*‐butyl ether:methanol = 3:1, v/v) were added, vortexed for 15 min. Then 200 µL water was added, followed by vortexing for 1 min. After centrifugation at 12 000 rpm for 10 min at 4 °C, 200 µL supernatant of each sample was transferred and concentrated to complete dryness, and then resuspended in 200 µL mobile phase B, vortexed for 3 min, centrifuged at 12 000 rpm for 3 min. The supernatants were transferred for LC‐MS/MS analysis. The data acquisition instrument system mainly includes UPLC and MS/MS. The chromatographic column is Thermo Accucore C30 column (2.1 × 100 mm, 2.6 µm).

### Measurement of Serum Parameters

To detect the degree of liver injury in mice by IR treatment, serum ALT and AST levels, two indicators of hepatocellular injury^[^ ^35^ ^]^ were measured using the Automated Biochemistry Analyze System (BS‐240VET, Mindray), according to the operating instruction.

### Liver NAD and NADH Measurement

Mice liver samples (10–30 mg) were homogenized and performed for NAD/NADH extraction using a commercially available kit (BC0315, Solarbio). Extracted homogenate was assayed at 570 nm by a 96‐well microplate reader (BIO‐RAD), according to the manufacturer's instructions.

### Histological Staining

Mice liver tissue samples were fixed overnight in 4% formalin, processed for paraffin embedding, and sectioned (5 µm per section). Tissue sections were stained with hematoxylin and eosin (H&E) and photographed by light microscopy. Areas of necrotic or imminent necrosis of tissue were identified by the presence of eosinopenia, loss of cell structure, vacuolization, cell rupture, or nucleolysis. To facilitate the analysis, necrotic areas with all features and areas about to undergo necrosis without nucleolysis but with most features were included in the quantitative analysis of necrosis. Immunohistochemistry staining was performed to analyze the expression profiles of specific proteins in liver tissue. Briefly, slides were de‐paraffinized, rehydrated, and boiled in a microwave for 10–12 min in 10 × 10^−3^ m citrate buffer or Tris‐EDTA buffer (pH 9.0). After incubation with 2% bovine serum albumin for 30 min at room temperature, liver sections were incubated with primary antibodies at 4 °C overnight, followed by incubation with horseradish peroxidase (HRP)‐conjugated secondary antibodies (ZLI‐9018, ZSGB‐BIO) for 2 h at room temperature. Specimens were washed three times then developed with the DAB substrate kit (ZLI‐9018, ZSGB‐BIO) and counterstained with hematoxylin. Antibodies used in this study are summarized in Key Resources Table.

### Quantitative Real‐Time PCR

Total RNA was extracted with Total RNA Extraction Reagent (R401‐01, Vazyme). cDNA was generated using HiScript II Q RT SuperMix for qPCR (R223‐01, Vazyme) following the manufacturer's instructions. Universal SYBR Green Fast qPCR Mix (RK21203, Abclonal) was used for qPCR on CFX Connect Real‐Time PCR Detection System (BIO‐RAD). mRNA levels were normalized to Gapdh according to the ^△△^Ct calculation method.

### Western Blot Assay

Total protein was isolated from tissue samples using RIPA lysis buffer (P0013E, Beyotime) with protease inhibitor cocktail tablets (HY‐K0011; MedChemExpress) and phosphatase inhibitor tablets (G2007, Servicebio). Cleared lysates were quantified using BCA Protein Assay Kit (P0012, Beyotime) and separated on SDS‐PAGE gels, transferred onto PVDF membranes (IPVH00010, Millipore). The membranes were blocked in 5% skim milk and incubated with primary antibodies at 4 °C overnight. HRP‐conjugated secondary antibodies were applied for 1 h at room temperature. Chemiluminescence was detected with commercial ECL reagents (E411‐03; Vazyme). Gapdh was used as a loading control. Antibodies used in this study are summarized in Key Resources Table.

### Statistical Analysis

Images in this study are representative of several experiments from different biological replicates independently. Statistical analyses were performed using GraphPad Prism 7 (GraphPad Software). Two‐tailed *P*‐values < 0.05 were considered significant (*P < 0.05, **P < 0.01, ***P < 0.001, and ****P < 0.0001, unless otherwise indicated).

## Conflict of Interest

The authors declare no conflict of interest.

## Author Contributions

B.X., P.Z., and X.T. contributed equally to this work. S.L. and D.Y.: conceptualization; B.X., P.Z., X.T., S.W., J.S., Y.Z., C.G., P.M., and C.Z.: investigation; B.X., P.Z., and D.Y.: writing‐original draft; B.X., P.Z., X.T., H.Q, S.L., and D.Y.: writing‐review and editing; P.Z., P.M., C.Z., S.L., and D.Y.: funding acquisition; and S.L. and D.Y.: supervision.

## Supporting information

Supporting Information

## Data Availability

The data that support the findings of this study are available from the corresponding author upon reasonable request.
