# Supplementary material for: Metabolic Rewiring of Kynurenine Pathway during Hepatic Ischemia–Reperfusion Injury Exacerbates Liver Damage by Impairing NAD Homeostasis
Source: Adv Sci (Weinh). 2022 Oct 30;9(35):2204697. doi: 10.1002/advs.202204697 (PMC9762284; doi:10.1002/advs.202204697)
Supplement: Supplementary file 1 — Supporting Information [file ADVS-9-2204697-s001.pdf]

ADVANCED SCIENCE
Open Access

Supporting Information

for *Adv. Sci.*, DOI 10.1002/advs.202204697

Metabolic Rewiring of Kynurenine Pathway during Hepatic Ischemia–Reperfusion Injury Exacerbates Liver Damage by Impairing NAD Homeostasis

Bowen Xu, Peng Zhang, Xiaolong Tang, Shiguan Wang, Jing Shen, Yuanwen Zheng, Chao Gao, Ping Mi, Cuijuan Zhang, Hui Qu, Shiyang Li* and Detian Yuan**

Supporting Information

Metabolic Rewiring of Kynurenine Pathway During Hepatic Ischemia-Reperfusion Injury Exacerbates Liver Damage by Impairing NAD Homeostasis

Bowen Xu, Peng Zhang, Xiaolong Tang, Shiguan Wang, Jing Shen, Yuanwen Zheng, Chao Gao, Ping Mi, Cuijuan Zhang, Hui Qu, Shiyang Li,* and Detian Yuan**

Supplementary Figure Legends

Supplementary Figure 1. Metabolic rewiring of the KP in the murine hepatic IR model. (A) Representative HE of the liver sections from mice subjected to 90-min of ischemia followed by different reperfusion durations. (B) Global sample distribution profiles and relationships analyzed by the hierarchical clustering. (C) Volcano plots indicating the differentially expressed metabolites (red, upregulated metabolites; blue, downregulated metabolites) in ischemia group relative to sham controls. THe metabolites in KP were highlighted. The vertical dashed gray lines in the plot represent log2 normalized fold change equal to 1 and -1. The horizontal dashed gray line represents false discovery rate (FDR) equal to 0.05. (A-B) n = 4 mice in each group.

Supplementary Figure 2. Afmid and Kyat2 are dramaticlly upregulated in the post-ischemic liver. (A) Representative IHC staining of Kmo, Kyat1 and Kyat3 in the liver sections from mice subjected to 90-min of ischemia and subsequent reperfusion for the indicated durations. (B) Representative IHC of KYAT2 in the liver sections of individuals subjected to hepatic IR surgery.

Supplementary Figure 3. Upregulation of Kyat2 is related to eIF2α phosphorylation in the ischemic livers. (A) Relative mRNA expression of *Afmid* and *Kyat2* in sham or ischemic livers. n = 4 mice per group. (B-C) The protein levels (B) and quantitative analysis (C) of Kyat2 and p-eIF2α in livers from mice subjected to sham, ischemia with 5 mg/kg ISRIB by i.p. or vehicle for 90-min.GAPDH served as a loading control. (D) The correlation of quantitative protein levels between Kyat2 and p-eIF2α. The correlations were evaluated with Spearman's test. (E) Representative IHC staining of Kyat2 and p-eIF2α in livers from mice subjected to sham, ischemia with 5 mg/kg ISRIB by i.p. or vehicle for 90-min using serial section. (B-E) n = 3 mice per group. (A,C) Paired student's *t* test was used. **P* < 0.05, ***P* < 0.01, ****P* < 0.001, *****P* < 0.0001. Data are presented as the mean ± SEM.

Supplementary Figure 4. Elevated oxidative stress in the livers subjected to hepatic IR surgery**.** (A) UPLC-MS/MS detection of a panal of oxidized fatty acids in ischemic livers and sham-operated controls. n = 4 mice per group. (B-D) Representative IHC staining of E06 (B), 4-HNE (C), and MDA (D) in sham and IR-treated livers. Paired student's t test was used. *P < 0.05, ***P* < 0.01, ****P* < 0.001, *****P* < 0.0001. Data are presented as the mean ± SEM.

Supplementary Figure 5. Kyat2 knockdown restores liver NAD and NADH levels and reduces lipid

peroxidation and inflammatory in the ischemic livers. (A) The short interfering RNAs (siRNAs) corresponding to three target sites of mouse Kyat2 coding region (indicated as siKyat2 #1, #2, #3, respectively) were designed and tested their targeting efficiency. The most two potent polymer #1 and #3 were mixed and used for in vivo experiments. (B) Cationic liposome-encapsulated siKyat2 and siCtrl were injected intravenously into the mice and hepatic IR was performed 48 hours later. The protein levels of Kyat2 in livers from siKyat2- or siCtrl-treated mice with 90-min ischemia were determined by immunoblotting. (C) Representative IHC staining of E06, MDA, 4-HNE in mice liver sections from (B). (D) Representative images of gross morphology of livers under IR treatment with siCtrl or siKyat2 injection for 24 h .(E) Serum ALT and AST levels in mice from (D). (F) Representative IHC staining of Ly6B and CD45 in mice liver tissue samples from (D). (G) Relative mRNA expression of *Il1a, Ilb, Ccl2, Ccl3, Tnf, Ccl4, Ccr1, Tgfb* in mice liver tissue samples from (D). (B-C) n = 4 mice in siCtrl group, n = 6 mice in siKyat2 group. (D-G) n = 6 mice per group. Paired student's *t* test was used. **P* < 0.05, ***P* < 0.01, ****P* < 0.001, *****P* < 0.0001. Data are presented as the mean ± SEM.

Supplementary Figure 6. Nicotinate phosphoribosyltransferase (Naprt) inhibitor 2-hydroxynicotinic acid (2-HNA) has a neglectable effect on hepatic IR-induced liver injury. (A) Representative images of gross morphology of livers from mice with 15 mg/kg 2-HNA or vehicle injection by i.p. for 24 h. (B-C) Survival probability and serum ALT/AST levels of mice from (A). (A-C) n = 3 mice per group. Paired student's *t* test was used. Data are presented as the mean ± SEM.

Supplementary Figure 7. FK866 enhances immune infiltration and inflammatory response in the livers subjected to hepatic IR insult. (A-B) IHC staining of Ly6B in sham (A) and hepatic IR-injured livers (B) treated with 15 mg/kg FK866 or vehicle. (C) Relative mRNA expression of *Tnf, Ccl2* and *Ccl4* in sham or IR-injured livers treated with FK866 or vehicle. **P* <0.05 by Student's t test. (A-C) n = 4 mice per group. Data are presented as the mean ± SEM.

Supplementary Figure 8. Lip-1 mitigates FK866-induced immune infiltration and inflammatory response in the lives subjected to hepatic IR insult. (A-B) IHC staining of Ly6B (A) and mRNA levels of *Il1a*, *Ccl3*, *Ccl4*, *Ccr1*, *Ccl2* and *Cxcl13* (B) in livers from IR-injured mice treated as indicated. n = 4 mice per group. Unpaired student's *t* test was used. **P* < 0.05, ***P* < 0.01, ****P* < 0.001. Data are presented as the mean ± SEM.

Supplementary Figure 9. NMN effectively alleviates immune infiltration and inflammatory response by IR injury. (A-B) IHC staining of CD45 (A) and relative mRNA expression of *Il33* and *Cxcl13* (B) in livers from IR-injured mice treated with NMN or vehicle. n = 4 mice per group. Unpaired student's *t* test was used. ***P* < 0.01, ****P* < 0.001. Data are presented as the mean ± SEM.

Supplementary Figure 1.

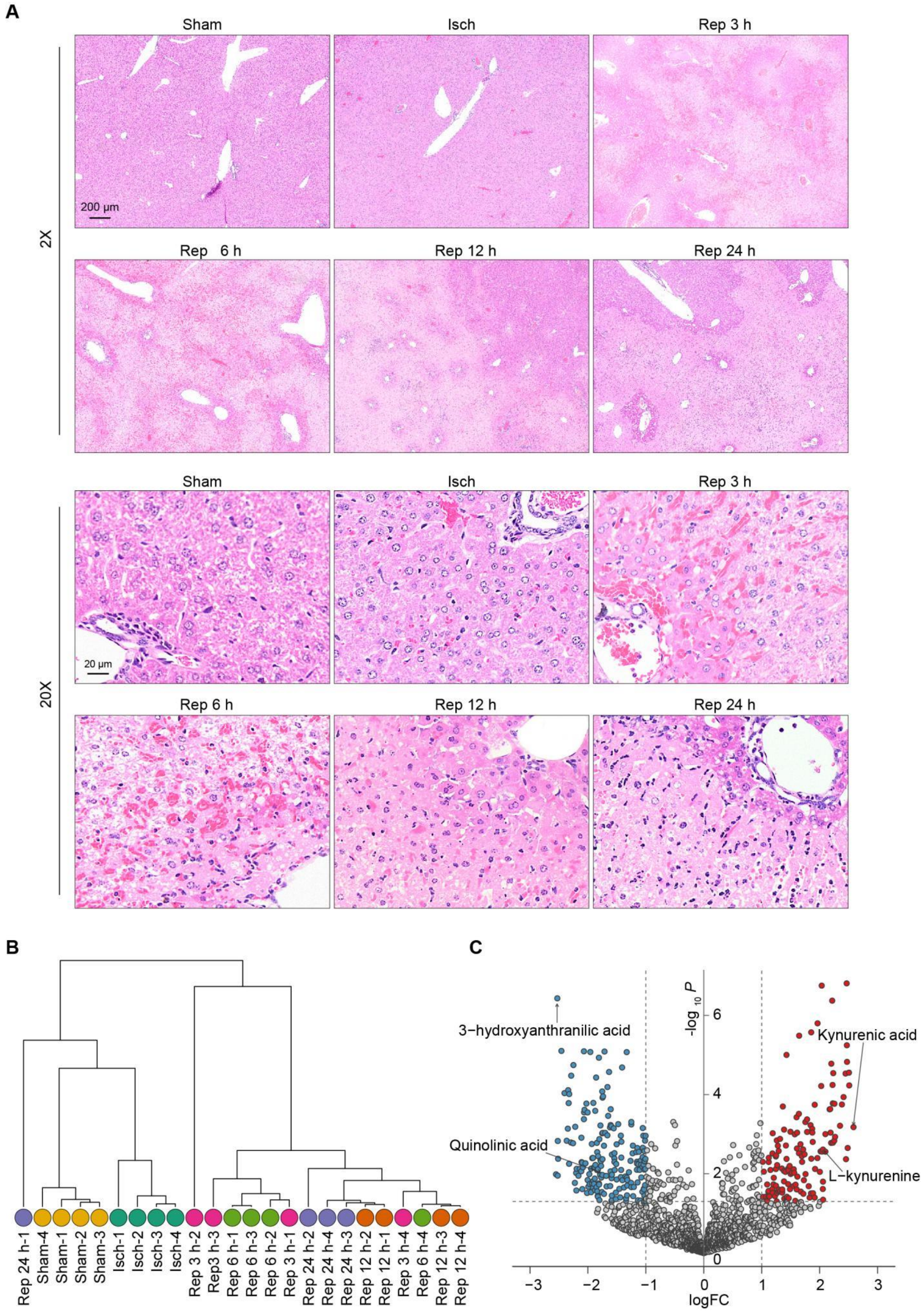

Supplementary Figure 2.

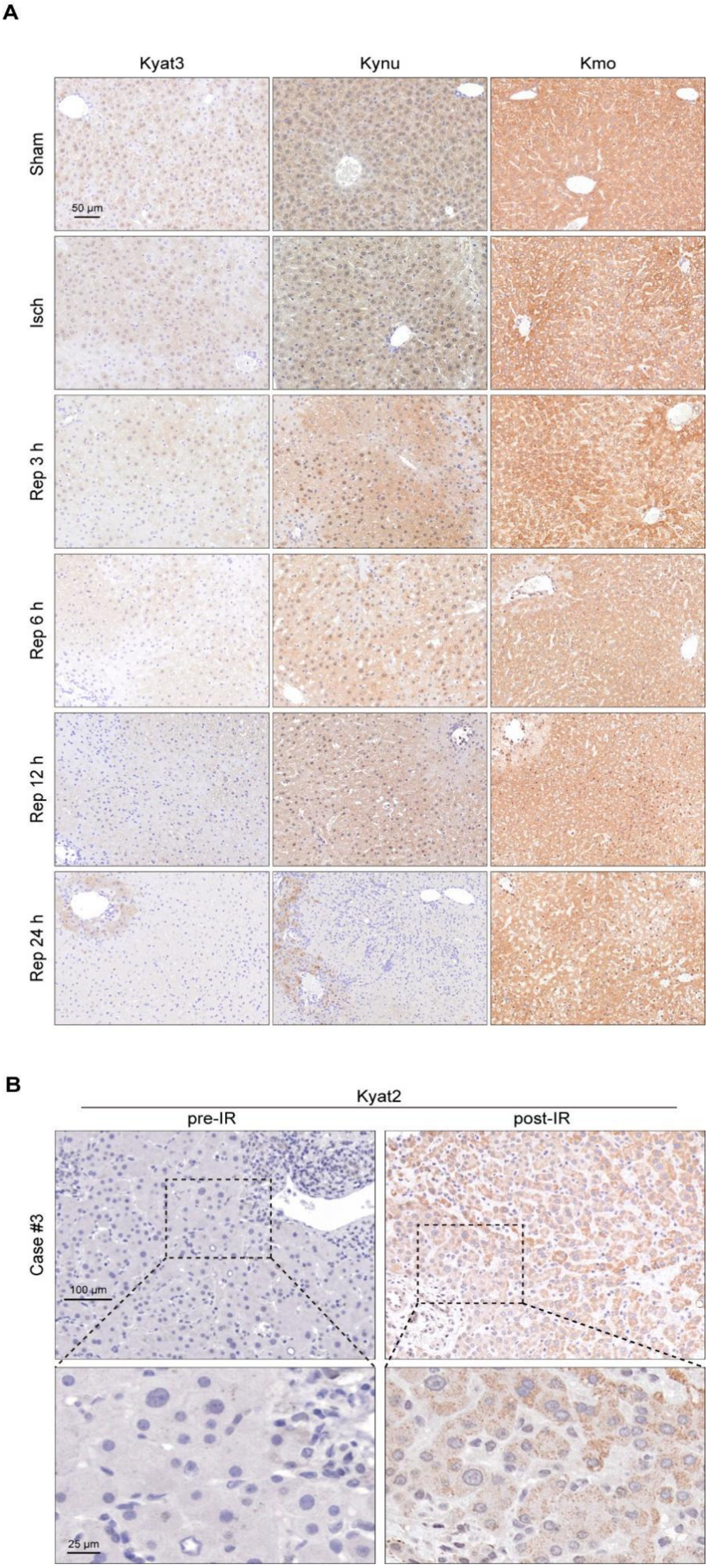

Supplementary Figure 3.

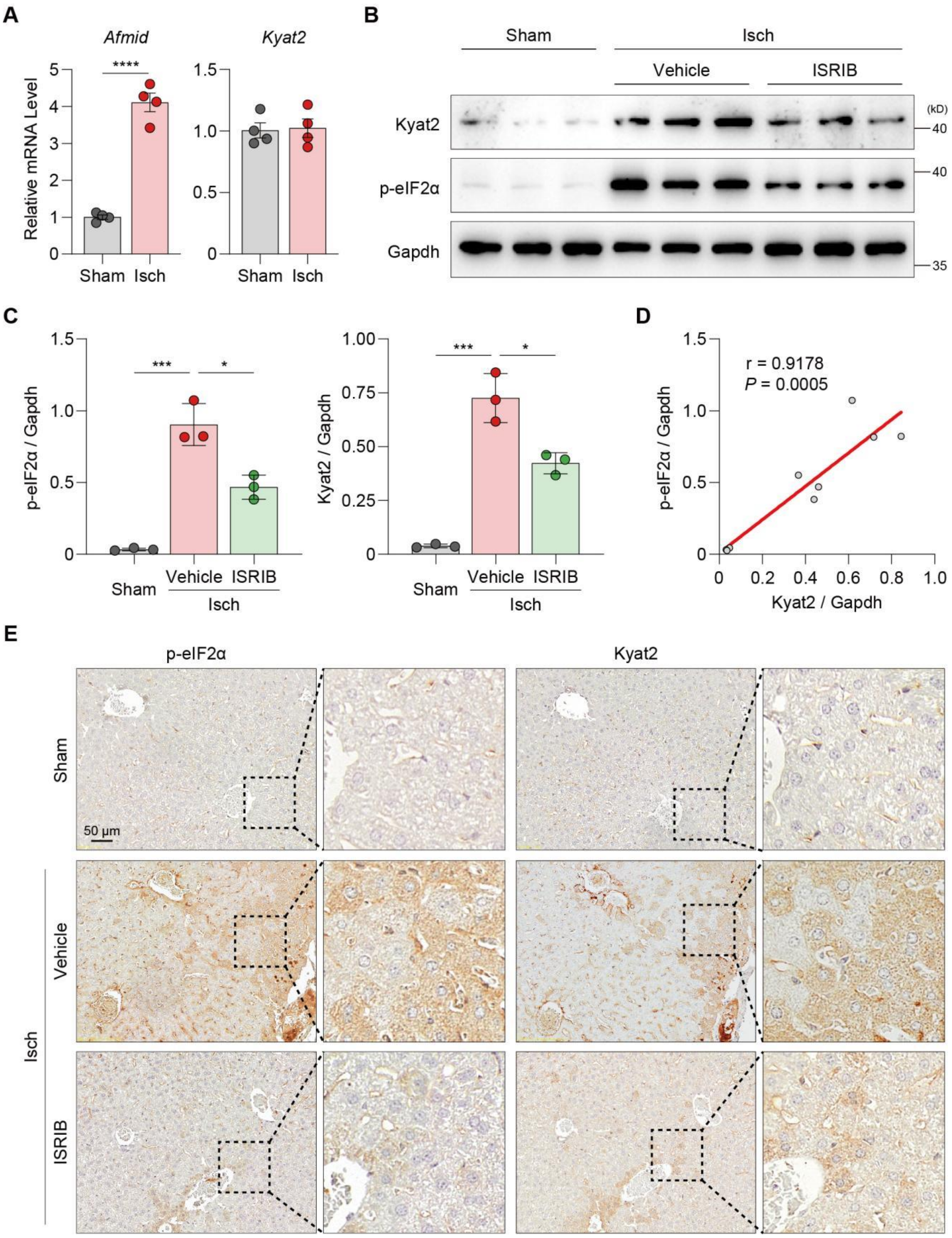

Supplementary Figure 4.

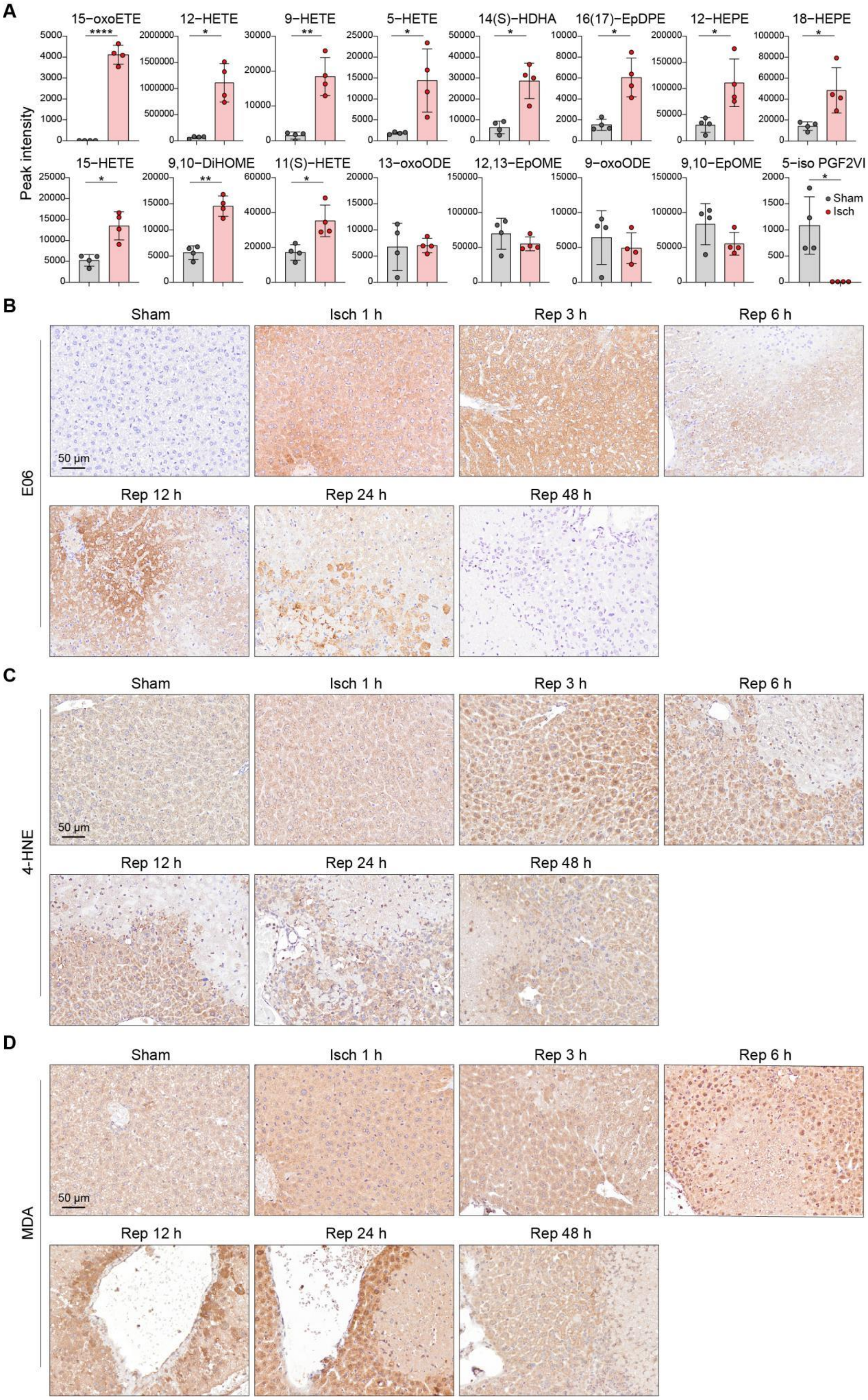

Supplementary Figure 5.

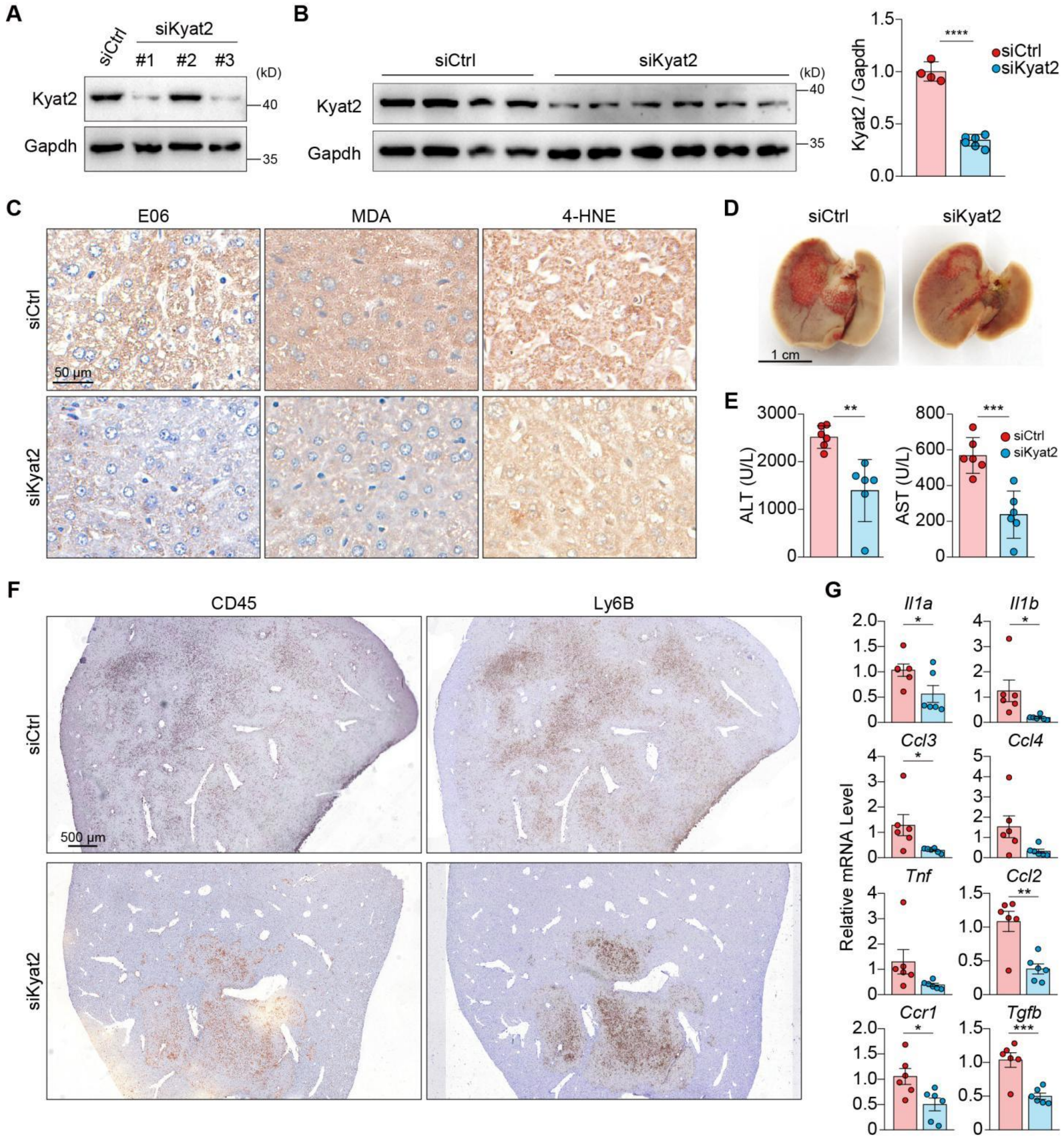

Supplementary Figure 6.

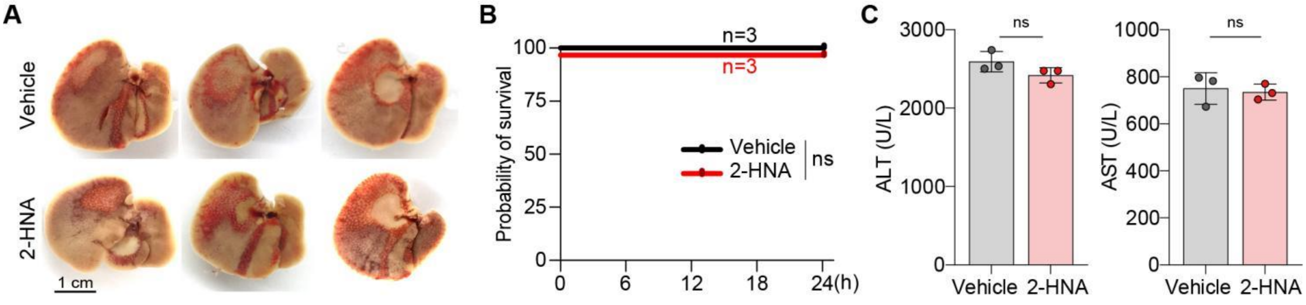

Supplementary Figure 7.

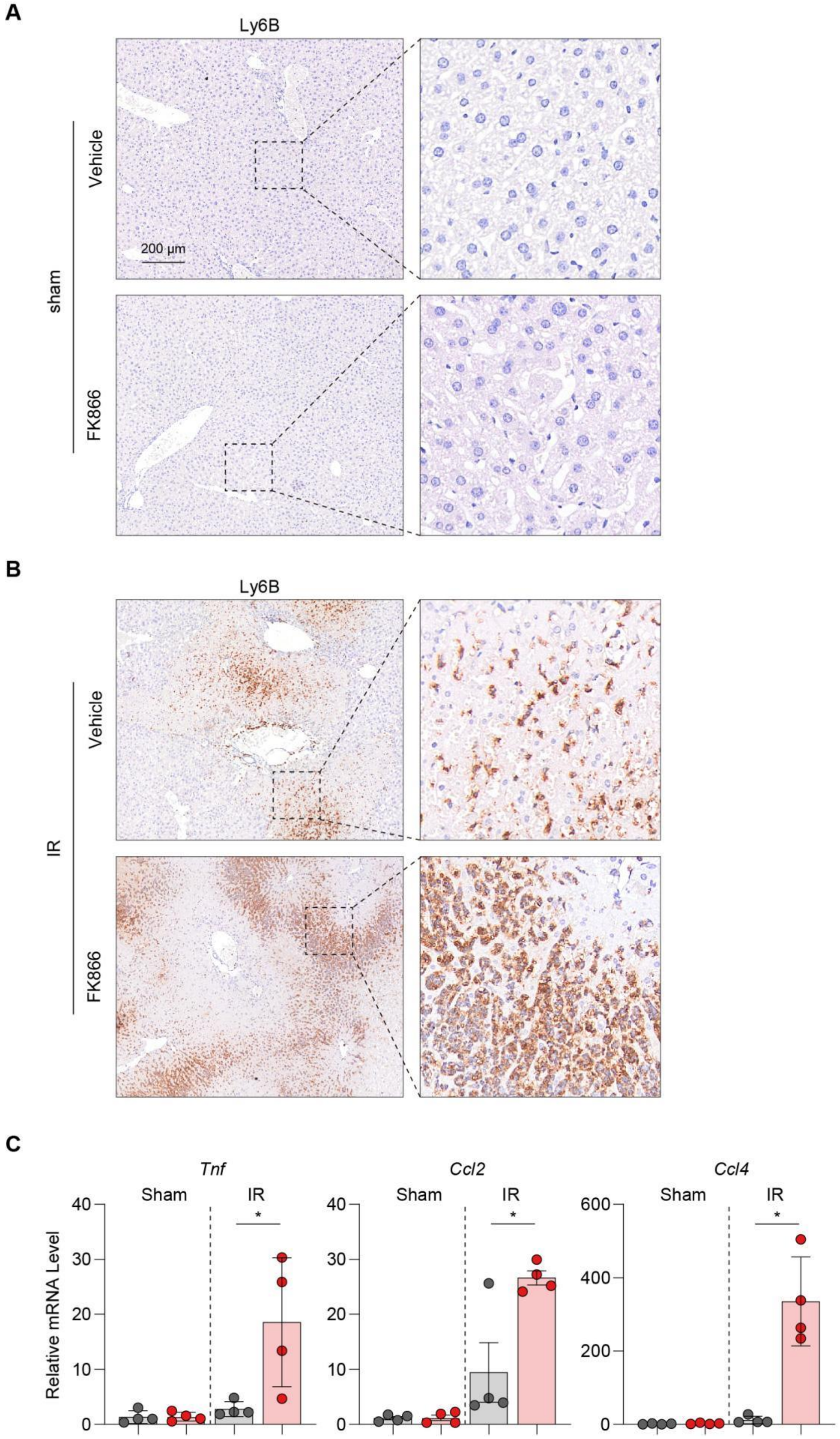

Supplementary Figure 8.

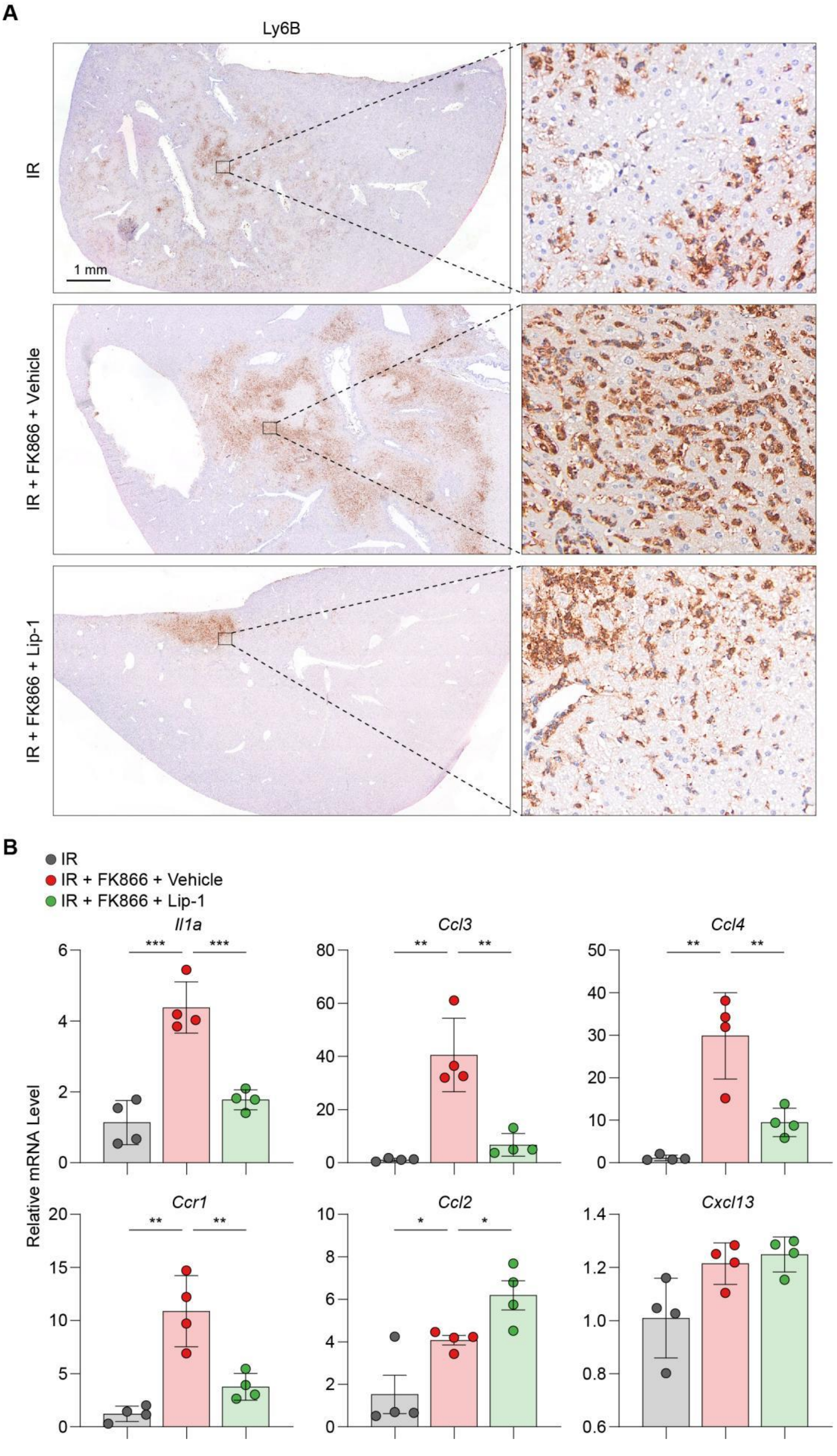

Supplementary Figure 9.

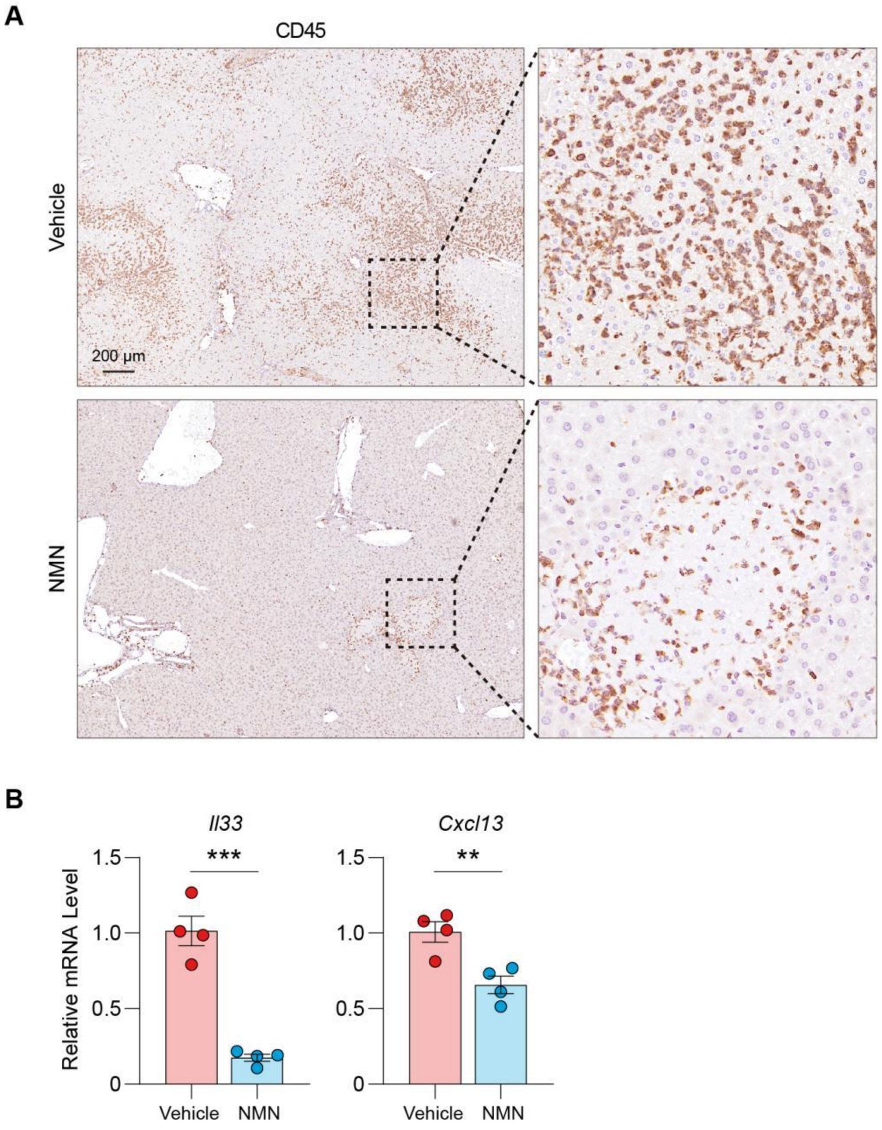

Supplementary Figure S.

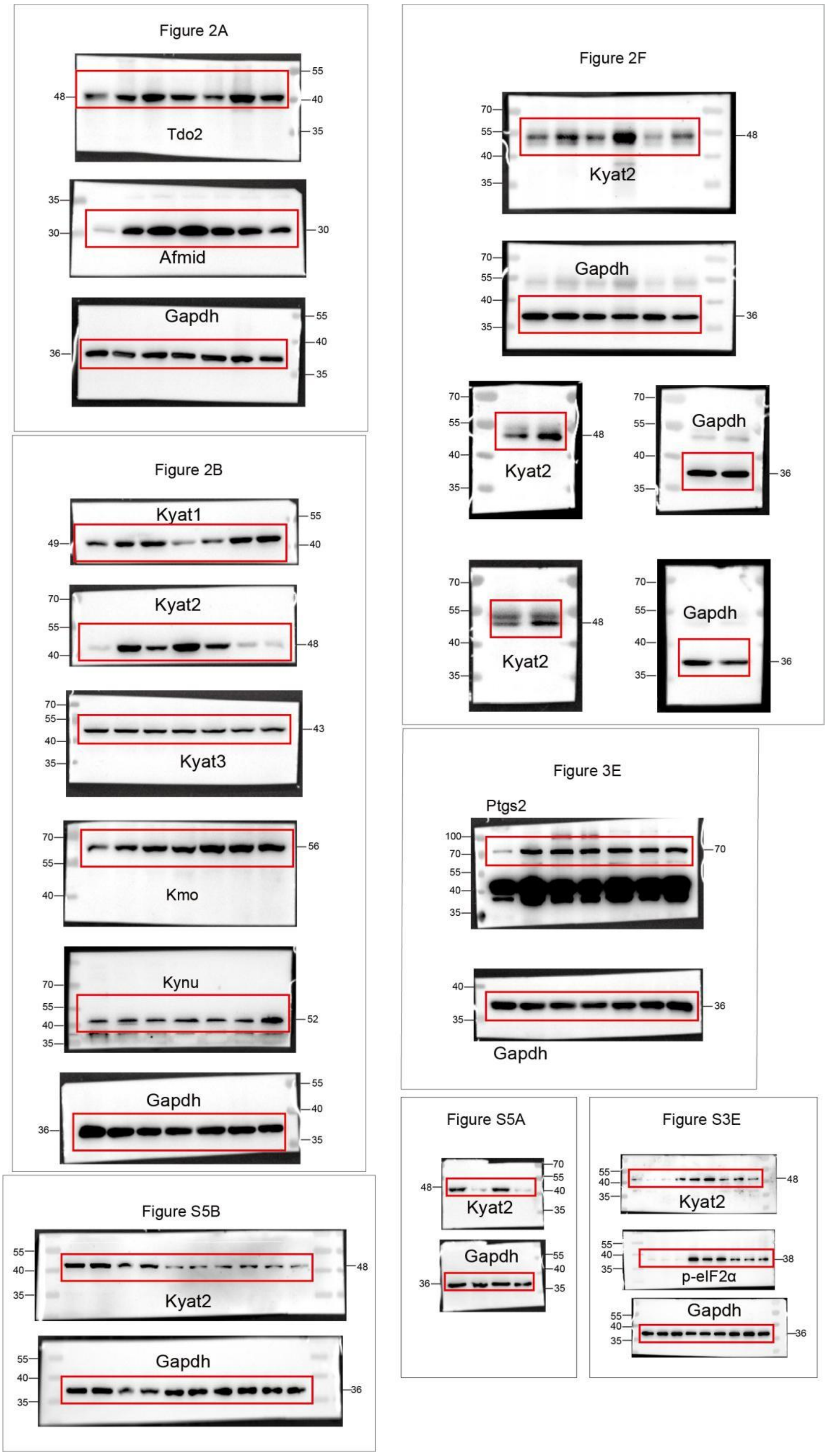

Supplementary Materials and Methods

Key Resources Table

REAGENT or RESOURCE	SOURCE	IDENTIFIER
**Antibodies**		
Aadat（Kyat2）	Proteintech	13031-1-AP
Kmo	Proteintech	10698-1-AP
Kyat3	Santa	sc-166922
TDO2	Proteintech	15880-1-AP
AFMID	Proteintech	19522-1-AP
MDA	ENZO	01072110
Ly6B	abcam	ab53457
E06	Avanti	330001S
Kynu	Proteintech	11796-1-AP
CD45	Servicebio	GB11066
Cox2	Abclonal	A1253
GAPDH	Abways	AB0037
4-Hydroxynonenal	Abcam	ab46545
Kyat1	Abcam	Ab194296
p-eiF2α	CST	#3398
**Experimental Models: Organisms/Strains**		
Mouse:C57BL/6J	Charles River Laboratories	Beijing, China
**Chemicals, and Recombinant Proteins**		
Liproxstatin-1	Selleck	S7699
FK866	Topscience	T2644
NMN	Topscience	T4721
2-HNA	Energy-Chemical	A010247
ISRIB	MedChemExpress	HY-12495
In vivo-jetPEI™	PolyPlus	201-10G

**Software and Algorithms**		
GraphPad prism	Graphpad	8.0.1
Image J	NIH/Macintosh	ImageJ2

**Oligonucleotides**	**Sequence**	**Supplier**
m_Il1α_F	CGAAGACTACAGTTCTGCCATT	Sangon Biotech
m_Il1α_R	GACGTTTCAGAGGTTCTCAGAG	Sangon Biotech
m_Il1b_F	GCAACTGTTCCTGAACTCAACT	Sangon Biotech
m_Il1b_R	ATCTTTTGGGGTCCGTCAACT	Sangon Biotech
m_ Tnf_F	CCCTCACACTCAGATCATCTTCT	Sangon Biotech
m_ Tnf_R	GCTACGACGTGGGCTACAG	Sangon Biotech
m_Ccl2_F	TTAAAAACCTGGATCGGAACCAA	Sangon Biotech
m_Ccl2_R	GCATTAGCTTCAGATTTACGGGT	Sangon Biotech
m_Ccl3_F	GCAACCAAGTCTTCTCAGCG	Sangon Biotech
m_Ccl3_R	TTGGACCCAGGTCTCTTTGG	Sangon Biotech
m_Ccl4_F	TTCCTGCTGTTTCTCTTACACCT	Sangon Biotech
m_Ccl4_R	CTGTCTGCCTCTTTTGGTC	Sangon Biotech

	AG	
m_Tgfb_F	CTCCCGTGG CTTCTAGTG C	Sangon Biotech
m_Tgfb_R	GCCTTAGTT TGGACAGGA TCTG	Sangon Biotech
m_Ccr1_F	CTGAGGGCC CGAACTGTT AC	Sangon Biotech
m_Ccr1_R	GGCTAGGGC CCAGGTGAT	Sangon Biotech
m_IL33_F	TCCAACTCC AAGATTTCC CCG	Sangon Biotech
m_IL33_R	CATGCAGTA GACATGGCA GAA	Sangon Biotech
m_Cxcl13_F	ATATGTGTG AATCCTCGT GCCA	Sangon Biotech
m_Cxcl13_R	GGGAGTTGA AGACAGACT TTTGC	Sangon Biotech
**siRNA**	**Sequence**	**Supplier**
m_siKyat2-1	cctaagaccttgat acagaat	GenePharma
m_siKyat2-3	ggtgaccgcaag aaggaaatc	GenePharma
